# Baicalin protects retinal ganglion cells by regulating microglial polarization and ferroptosis via the JAK2/STAT3 pathway

**DOI:** 10.3389/fphar.2025.1660294

**Published:** 2025-10-24

**Authors:** Huan Yu, Tianzhao Xu, Qiaozong Feng, Yilin Wang, Jun Zou, Xiaoyun Ma

**Affiliations:** ^1^ Shanghai University of Medicine and Health Sciences Affiliated Zhoupu Hospital, Shanghai, China; ^2^ Shanghai Pudong New Area Hangtou Hesha Community Health Service Center, Shanghai, China; ^3^ Shanghai University of Medicine and Health Sciences, Shanghai, China; ^4^ Department of Ophthalmology, Shanghai Tenth People’s Hospital, Tongji University, Shanghai, China

**Keywords:** baicalin, retinal ganglion cells, microglia, iron death, oxidative stress

## Abstract

**Introduction:**

This study investigated the protective effects of baicalin on retinal ganglion cells (RGCs) subjected to ischemia-reperfusion injury and explored the underlying mechanisms.

**Methods:**

By establishing an *in vitro* model of oxygen-glucose deprivation/reperfusion (OGD/R) in a microglia-RGC co-culture system, we examined the protective role of baicalin against RGC injury.

**Results:**

The results demonstrated that baicalin significantly suppressed the M1 polarization of microglia, promoted their shift toward M2 polarization, and alleviated oxidative stress and ferroptosis in RGCs by inhibiting the JAK2/STAT3 signaling pathway.

**Conclusion:**

This study reveals that baicalin protects RGCs by modulating microglial polarization and inhibiting ferroptosis, providing novel insights for the treatment of glaucoma.

## Introduction

Glaucoma is the leading cause of irreversible blindness worldwide, affecting over 79 million people (Chen et al., 2025). In China, the prevalence of glaucoma exceeds 2.5% and is expected to rise significantly with the aging of the population (Publisher Correction GBD, 2019 Blindness and Vision Impairment Collaborators, 2024; Yuan et al., 2024). The irreversible loss of retinal ganglion cells (RGCs) represents the core pathological feature of glaucoma. Elevated intraocular pressure (IOP) induces ischemia-reperfusion injury, which serves as a primary driver of RGC damage in glaucoma. This process begins with an acute IOP surge, which severely reduces blood flow to the optic disc and causes ischemia. Subsequently, when the IOP is abruptly lowered, blood flow resumes, leading to a rapid increase in retinal perfusion. This reperfusion, however, paradoxically triggers ischemia-reperfusion injury, further exacerbating RGC death (Fernández-Albarral et al., 2024; Chen et al., 2017). Current therapies for glaucoma are aimed at lowering IOP; while these treatments can slow disease progression, effective interventions targeting the core pathological process of RGC death are lacking (Storgaard et al., 2021).

Recent studies have shown that ischemia-reperfusion injury caused by elevated IOP can exacerbate the damage to RGCs. After pathological injury due to high IOP, abnormal accumulation of Fe2+ occurs, leading to an imbalance in glutathione metabolism and lipid peroxidation, ultimately inducing ferroptosis in RGCs (Yao et al., 2023; Zhang et al., 2024; Feng et al., 2024). Additionally, ischemia-reperfusion injury promotes the polarization of microglia toward the pro-inflammatory M1 phenotype, releasing neurotoxic factors (Wang Q. et al., 2024). These dual mechanisms provide new targets for neuroprotective therapies in glaucoma. Notably, baicalin, an active compound derived from the traditional Chinese herb *Scutellaria baicalensis*, has demonstrated multitarget neuroprotective effects (Tan et al., 2022; Ahmadi et al., 2022; Baradaran et al., 2021). This compound has shown therapeutic potential in various disease contexts, including diabetes, cancer, and cardiovascular disorders (Wang J. et al., 2024; Dinda et al., 2023; Xin et al., 2020). In ischemic brain models, baicalin was found to inhibit ferroptosis by modulating the Nrf-2 pathway (Liu et al., 2024) and to regulate microglial M1/M2 polarization, thereby reducing neuroinflammation (Li H. et al., 2021). Additionally, *in vitro* co-culture experiments have confirmed that baicalin alleviates oxidative stress, inflammation, and neuronal apoptosis induced by ischemia-reperfusion, by upregulating brain-derived neurotrophic factor (Li C. et al., 2021). Previous studies by our research group have demonstrated that baicalin can effectively protect rat RGCs from oxidative stress injury induced by glutamate (Yu et al., 2024).

Of particular interest is the JAK/STAT signaling pathway, which serves as a critical link between inflammatory responses and cell death. Under normal conditions, the activity of this pathway is barely detectable in the retina. However, acute IOP elevation triggers JAK/STAT activation in the inner nuclear layer and ganglion cell layer of the retina, which includes RGCs (Yu et al., 2021; Huang et al., 2007). Whether baicalin can protect RGCs by modulating ferroptosis and microglial polarization through the JAK/STAT signaling pathway remains unclear.

This study is the first to explore the neuroprotective effects of baicalin in the context of glaucoma. We constructed an *in vitro* oxygen-glucose deprivation/reperfusion (OGD/R) model to mimic the key pathological process of glaucoma. In addition, we performed a series of experiments to assess the protective effects of baicalin in RGCs and elucidated its dual mechanism of action through the JAK/STAT signaling pathway. By simultaneously targeting ferroptosis and microglial polarization, this multitarget therapeutic strategy not only offers new insights for neuroprotection in glaucoma but also provides a theoretical basis for investigating the mechanisms of bioactive compounds in traditional Chinese medicine.

## Materials and methods

### Cell culture

The R28 retinal progenitor cell line, derived from Sprague-Dawley rat retinas, is an immortalized adherent cell line used for *in vitro* studies on neuroprotection, cytotoxicity, and physiological functions of RGCs (Seigel, 2014). R28 cells (YaJi Bio, China) were cultured in Dulbecco-modified Eagle medium (Servicebio, China) supplemented with 10% fetal bovine serum (Servicebio, China) and 1% penicillin/streptomycin (Servicebio, China). The cells were maintained in a humidified incubator at 37 °C with 5% CO_2_. HAPI cells (YaJi Bio, China), a rat microglial cell line, were cultured under identical conditions.

### Establishment of the OGD/R model and baicalin intervention

R28 cells were seeded in the lower chambers of Transwell inserts (Corning, China), while HAPI cells were seeded in the upper chambers. After overnight culture in complete medium, the cells were subjected to OGD/R. The incubator atmosphere was adjusted to 95% N_2_ and 5% CO_2_, and glucose-free medium (premixed with baicalin) was used to simulate ischemic conditions. The co-culture system was incubated for 6 h. Following ischemic treatment, the medium was replaced with glucose-containing medium (premixed with baicalin) under normal oxygen conditions to simulate reperfusion, and the cells were cultured for an additional 24 h. The OGD/R models were treated with baicalin at concentrations of 10 μM, 50 μM, and 100 μM, with corresponding controls.

### Cell counting kit-8 assay

Post-intervention HAPI cells were collected, adjusted to a specific density, and seeded into 96-well plates (3 × 10^3^ cells/well). After 24 h of culture, the medium was replaced with 90 μL of fresh medium and 10 μL of Cell counting kit-8 (CCK-8) solution (Servicebio, China). The cells were incubated at 37 °C for 2 h, and their optical density (OD) values were measured at 450 nm by using a microplate reader (Huadong, China).

### Apoptosis detection

Cells were digested with trypsin (Servicebio, China) for 5 min at 37 °C, and the digestion was terminated by adding Dulbecco modified Eagle medium/Nutrient Mixture F-12 (DMEM-F12) medium containing fetal bovine serum. The cells were then centrifuged at 1,500 rpm for 3 min, and the supernatant was discarded. The cells were resuspended in phosphate-buffered saline (PBS), centrifuged again under the same conditions, and finally resuspended in binding buffer. A portion of the cells was stained with 0.4% trypan blue for 5 min at room temperature, and cell counts were performed under a microscope. The remaining cells were adjusted to a density of 1 × 10^5^ cells/mL in binding buffer. Annexin V (5 μL) and propidium iodide (5 μL; Beyotime, China) were added to 100 μL of cell suspension, and the cells were incubated for 15 min at room temperature in the dark. The volume was adjusted to 500 μL with binding buffer, and the cells were analyzed using flow cytometry.

### Reactive oxygen species detection

The fluorescence probe for reactive oxygen species (ROS), 2′−7′-dichlorodihydrofluorescein diacetate (DCFH-DA; Beyotime, China) was diluted with serum-free medium at a ratio of 1:1000, to achieve a final concentration of 10 μM. Post-intervention cells were washed twice with PBS. The cell culture medium was removed, and 1 μL of the DCFH-DA probe diluted in 1 mL complete medium was added. The cells were incubated at 37 °C for 30 min and then washed 3 times with serum-free cell culture solution to fully remove any DCFH-DA that did not enter the cells. After further incubation for 15 min at room temperature, the cells were washed 3 times with PBS. The staining of the cells was observed under a fluorescence microscope.

### Malondialdehyde assay

Cells were lysed with an appropriate volume of lysis buffer and incubated on ice for 30 min, with periodic pipetting. The lysate was centrifuged at 12,000 rpm for 10 min at 4 °C, and the supernatant was collected. Malondialdehyde (MDA) levels were measured by adding 0.2 mL of MDA working solution (Beyotime, China) to each sample. After being heated to 100 °C for 15 min, the samples were cooled and centrifuged at 2,000 rpm for 10 min. Absorbance was measured at 532 nm by using a microplate reader, and MDA levels were calculated using the OD values.

### Enzyme-linked immunosorbent assay

Cell culture medium was collected for enzyme-linked immunosorbent assay (ELISA; Solarbio, China). We set standard and sample wells and added 50 μL of standard products at different concentrations to each standard well according to the manufacturer’s instructions. A blank well was used as a control, without any sample or enzyme-labeled reagent (the other steps were the same). Next, 40 μL of diluent was added to the sample wells on the enzyme-labeled plate, and then, the sample was added to the bottom of the enzyme-labeled well, while trying not to touch the well walls, and gently shaken to mix. The enzyme-labeled reagent (100 μL per well) was added to each well, except for the blank well. The plate was sealed with a sealing film and incubated at 37 °C for 60 min. The seal film was carefully removed, and the liquid was discarded. The plate was shaken dry, and each well was filled with a washing liquid, which was left in place for 30 s, and then discarded. The washing step was repeated 5 times, and then, the wells were patted dry. Color developer A (50 μL) was added to each well, followed by color developer B (50 μL). The wells were gently shaken in the dark for 15 min at 37 °C. Termination solution (50 μL) was added to each well to terminate the reaction. The samples were analyzed within 15 min after the addition of the termination solution. The absorbance (OD values) of each well was measured at 450 nm, with blank hole zeroing.

### Western blot

Cells were lysed with radioimmunoprecipitation assay buffer (Servicebio, China), and protein concentrations were determined using the bicinchoninic acid method (Servicebio, China). Sodium dodecyl sulfate-polyacrylamide gel electrophoresis (SDS-PAGE) was performed using a standard procedure (Abe et al., 2017). In brief, 15 μL of the sample was loaded onto each well of the SDS-PAGE gel. After loading, electrophoresis was run at a constant voltage of 140 V until the bromophenol blue dye front reached the end of the gel. After electrophoresis, the protein samples were transferred to a nitrocellulose membrane (Millipore, United States), and subsequently blocked with 5% bovine serum albumin on a shaker at 70 rpm for 90 min at room temperature. After that, the membranes were incubated overnight with the primary antibodies at 4 °C. The following primary antibodies were used: anti-inducible nitric oxide synthase (iNOS) antibody (Affinity, China; 1:1000, Cat. No. AF0199), anti-arginase 1 (Arg1) antibody (Affinity, China; 1:1000, Cat. No. DF6657), anti-cleaved caspase 3 antibody (Servicebio, China; 1:1000, Cat. No. GB11532-50), anti-BCL2 antibody (Servicebio, China; 1:2000, Cat. No. GB153375-50), anti-p-JAK2 antibody (Affinity, China; 1:500, Cat. No. AF3024), anti-JAK2 antibody (Affinity, China; 1:1000, Cat. No. AF6022), anti-p-STAT3 antibody (Affinity, China; 1:500, Cat. No. AF3293), anti-STAT3 antibody (Affinity, China; 1:1000, Cat. No. AF6294), and anti-GAPDH antibody (Servicebio, China; 1:5000, Cat. No. GB15004-100). Goat anti-rabbit secondary antibodies were also used (Servicebio, China; 1:10000, Cat. No. GB23204). The nitrocellulose membranes were washed 3 times with Tris-buffered saline supplemented with Tween 20 (TBST) for 10 min each time, and the corresponding secondary antibody diluted in 1% TBST was added to the membranes, which were then incubated for 90 min at 37 °C. Signals were detected using an enhanced chemiluminescence kit and quantified using ImageJ software, with the results normalized to β-actin expression levels.

### Fe^2+^ and total Fe content measurement

Cells were lysed, and supernatants were collected after centrifugation at 1,500 rpm. Standards and samples were prepared in duplicate. For Fe^2+^ detection, an assay buffer was added, while for total iron detection, an iron reducer was added. After incubation at 37 °C for 30 min, an iron probe solution was added, and the samples were incubated for an additional 60 min in the dark. Absorbance was measured at 593 nm, and the Fe^2+^ and total Fe concentrations were calculated. The relative ratio of Fe^2+^ to total Fe was determined.

### Statistical analysis

SPSS software (version 27.0) was used for statistical analysis. Two-tailed Student *t*-tests were used to compare two groups, and one-way analysis of variance was used to compare 3 groups. Differences with *P* < 0.05 were considered statistically significant. All variables are presented as mean ± standard deviation.

## Results

### Baicalin enhances RGC activity and inhibits RGC apoptosis

**FIGURE 1:**
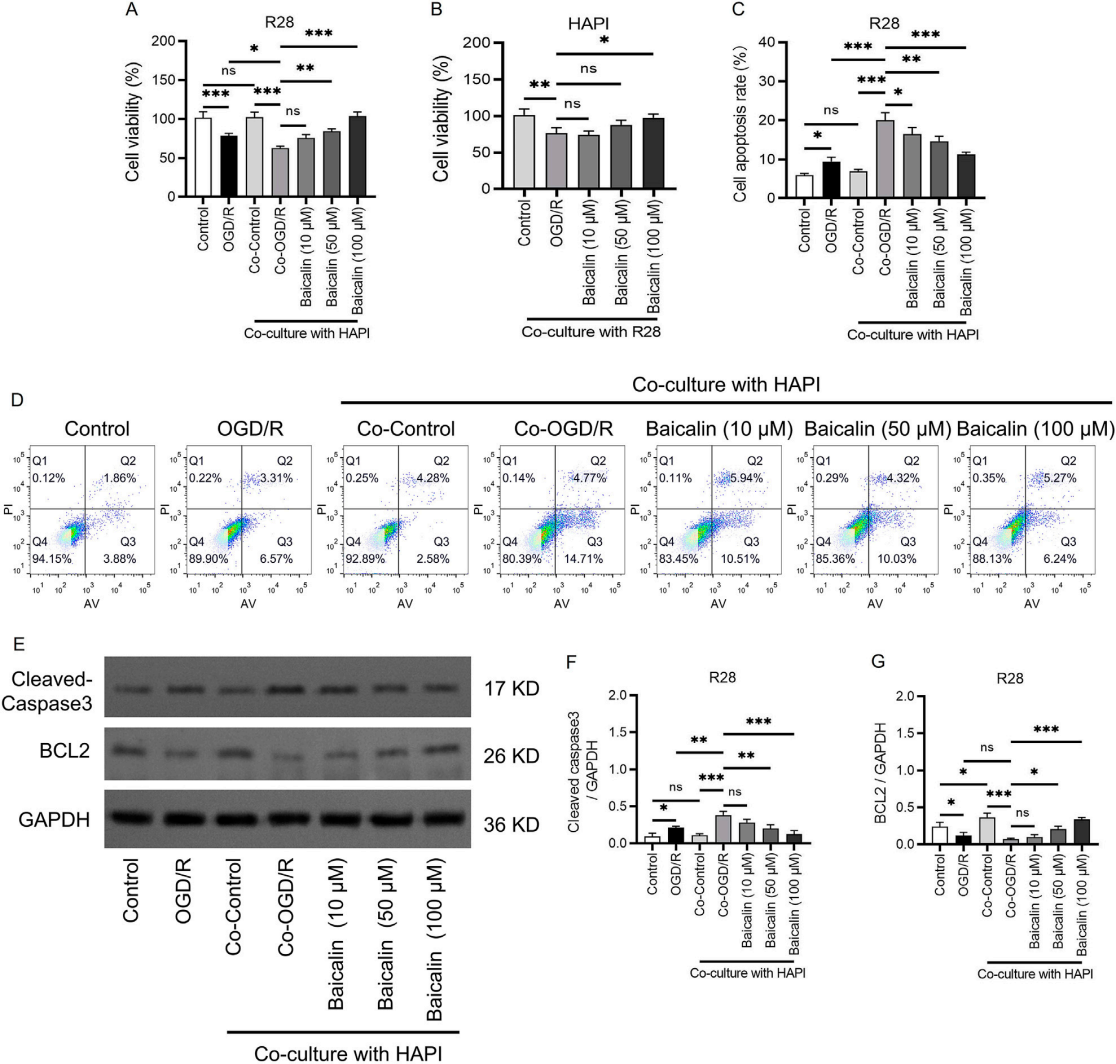
Baicalin enhances RGC activity and suppresses RGC apoptosis. **(A)** Viability of R28 cells. **(B)** Viability of HAPI cells. **(C,D)** Apoptosis rates of cells in different groups analyzed using apoptosis assays. **(E–G)** The protein levels and quantification of caspase 3 and BCL2. Mean ± SD; *n* = 3/group; *P < 0.05, **P < 0.01, ***P < 0.001. One-way analysis of variance. RGC, retinal ganglion cell.

To investigate the potential protective effects of baicalin on RGC and microglia co-culture models, we assessed cell viability using the CCK-8 assay. The results showed that compared to the normal control group, the OGD/R group exhibited significantly reduced cell viability, indicating that ischemia-reperfusion severely damaged R28 cells (Figure 1A). In the Co-OGD/R group, cell viability decreased to 0.522 ± 0.017, suggesting that the RGC and microglia co-culture conditions exacerbated RGC injury. Notably, there was no significant difference in cell viability between the 10 μM baicalin group and the OGD/R group, but cell viability was significantly higher in the 50 μM group and especially the 100 μM group than in the OGD/R group (Figure 1B), suggesting that baicalin can reduce cell injury caused by ischemia-reperfusion and has a protective effect on the co-culture model of RGCs and HAPI cells.

**FIGURE 2:**
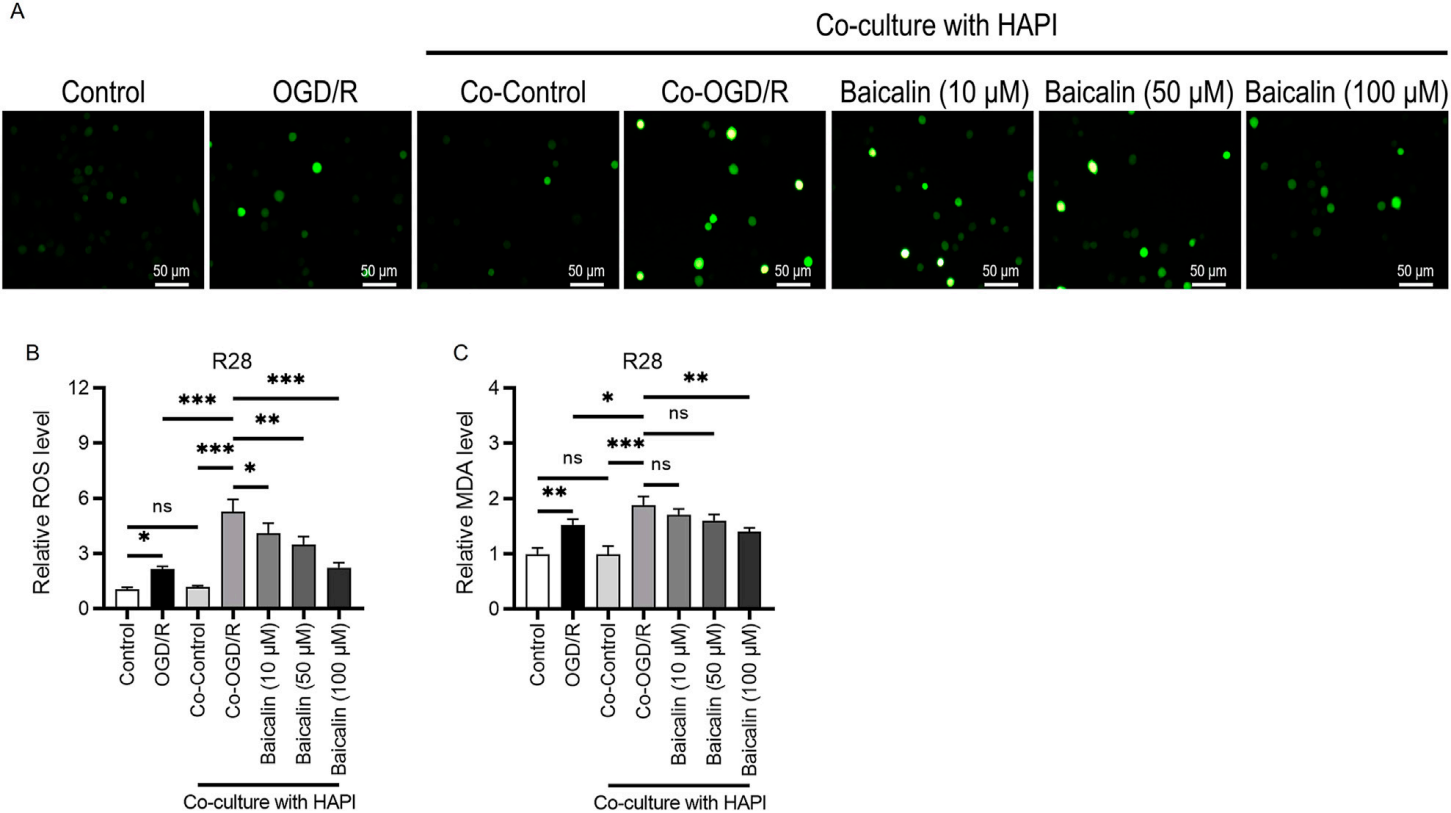
Baicalin mitigates oxidative stress in RGCs. **(A,B)** ROS fluorescence intensity in R28 cells in different groups. Scale bar: 50 μm. **(C)** MDA levels in R28 cells. Mean ± SD; *n* = 3/group; *P < 0.05, **P < 0.01, ***P < 0.001. One-way analysis of variance. RGCs, retinal ganglion cells; ROS, reactive oxygen species; MDA, malondialdehyde.

To further determine the safe therapeutic concentration and protective effects of baicalin, we conducted apoptosis assays (Figures 1C,D). The results revealed a significant increase in apoptosis rates in the OGD/R group as compared to the control group. Apoptosis rates in the Co-OGD/R group were markedly elevated as compared to the control group, suggesting that microglia may exacerbate apoptosis through the release of pro-inflammatory factors. Importantly, after treatment with baicalin, the apoptosis rates were significantly decreased. Additionally, Western blot experiments validated the above anti-apoptotic effects of baicalin. Baicalin treatment inhibited the pro-apoptotic protein caspase 3 and upregulated the anti-apoptotic protein BCL2, indicating that baicalin effectively reversed OGD/R and co-culture–induced apoptosis in a concentration-dependent manner (Figures 2E–G).

### Baicalin attenuates oxidative stress in RGCs

To evaluate the effect of baicalin on oxidative stress, we performed assays with DCFH-DA fluorescence probes and measured MDA levels. The results showed that both ROS fluorescence intensity (Figures 2A,B) and MDA levels (Figure 2C) were elevated in the OGD/R group, indicating oxidative stress-induced damage in R28 cells caused by ischemia-reperfusion. This effect was further exacerbated in the Co-OGD/R group. After baicalin intervention, oxidative stress markers showed a concentration-dependent decrease, with the 100 μM group exhibiting the most significant antioxidant effects. This suggests that baicalin alleviates oxidative stress injury caused by ischemia-reperfusion and protects RGCs by modulating oxidative stress-related pathways.

### Baicalin reduces inflammatory responses

**FIGURE 3:**
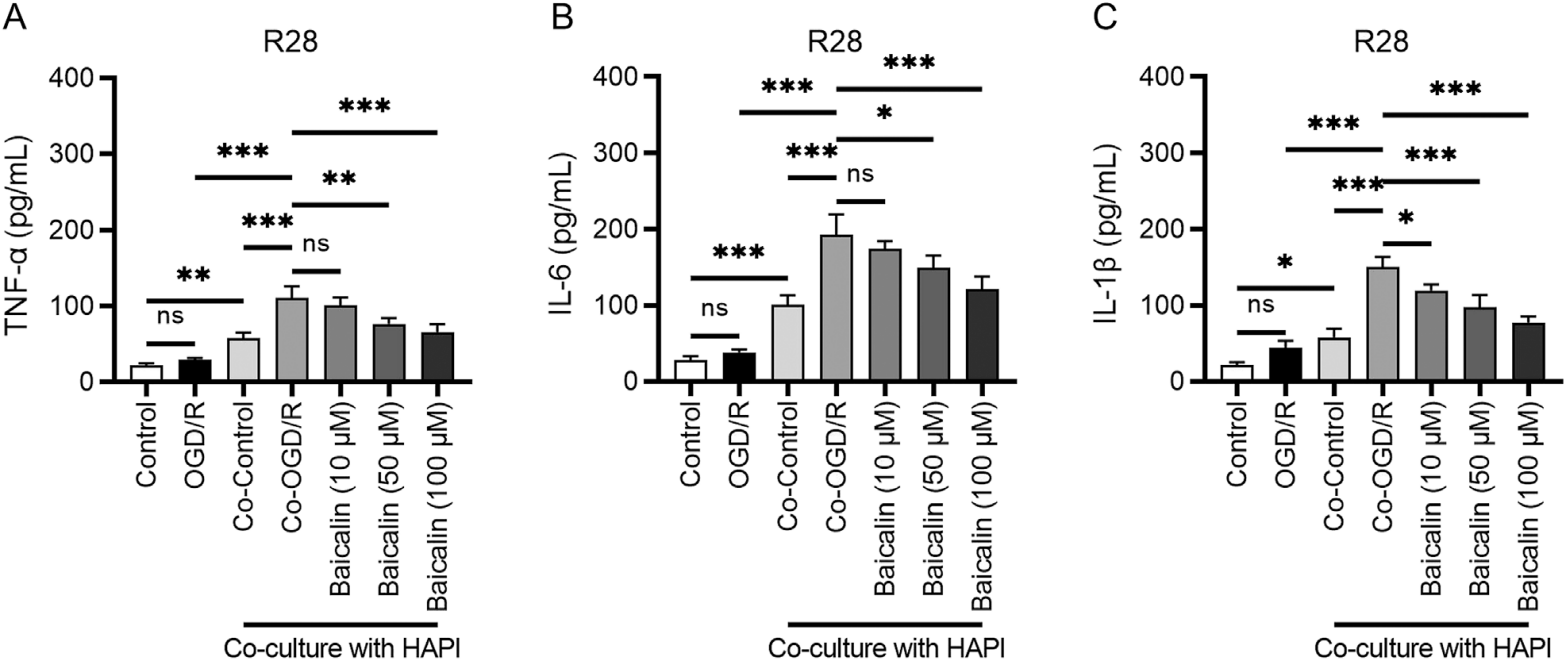
Baicalin inhibits inflammatory responses. **(A–C)** Levels of TNF-α, IL-6, and IL-1β in the normal control group, OGD/R group, Co-OGD/R group, and baicalin groups with different concentrations of baicalin. Mean ± SD; *n* = 3/group; *P < 0.05, **P < 0.01, ***P < 0.001. One-way analysis of variance. TNF-α, tumor necrosis factor-α; IL, interleukin; OGD/R, oxygen-glucose deprivation/reperfusion.

Research has shown that oxidative stress can stimulate the production of numerous inflammatory factors, which exacerbate neuronal cell death (Ryu et al., 2021; Scheid et al., 2021). Our ELISA results indicated that OGD/R treatment significantly increased the levels of tumor necrosis factor-α (TNF-α), interleukin (IL)-6, and IL-1β, confirming the successful induction of an inflammatory response. In the co-culture system of microglia and RGCs (Co-OGD/R group), the levels of these inflammatory factors were further elevated, suggesting that microglia significantly intensify the inflammatory response through intercellular interactions (Figures 3A–C). Baicalin treatment effectively inhibited the inflammatory response induced by OGD/R and co-culture, which may be related to the blocking of microglial polarization.

### Baicalin lightens microglial polarization

**FIGURE 4:**
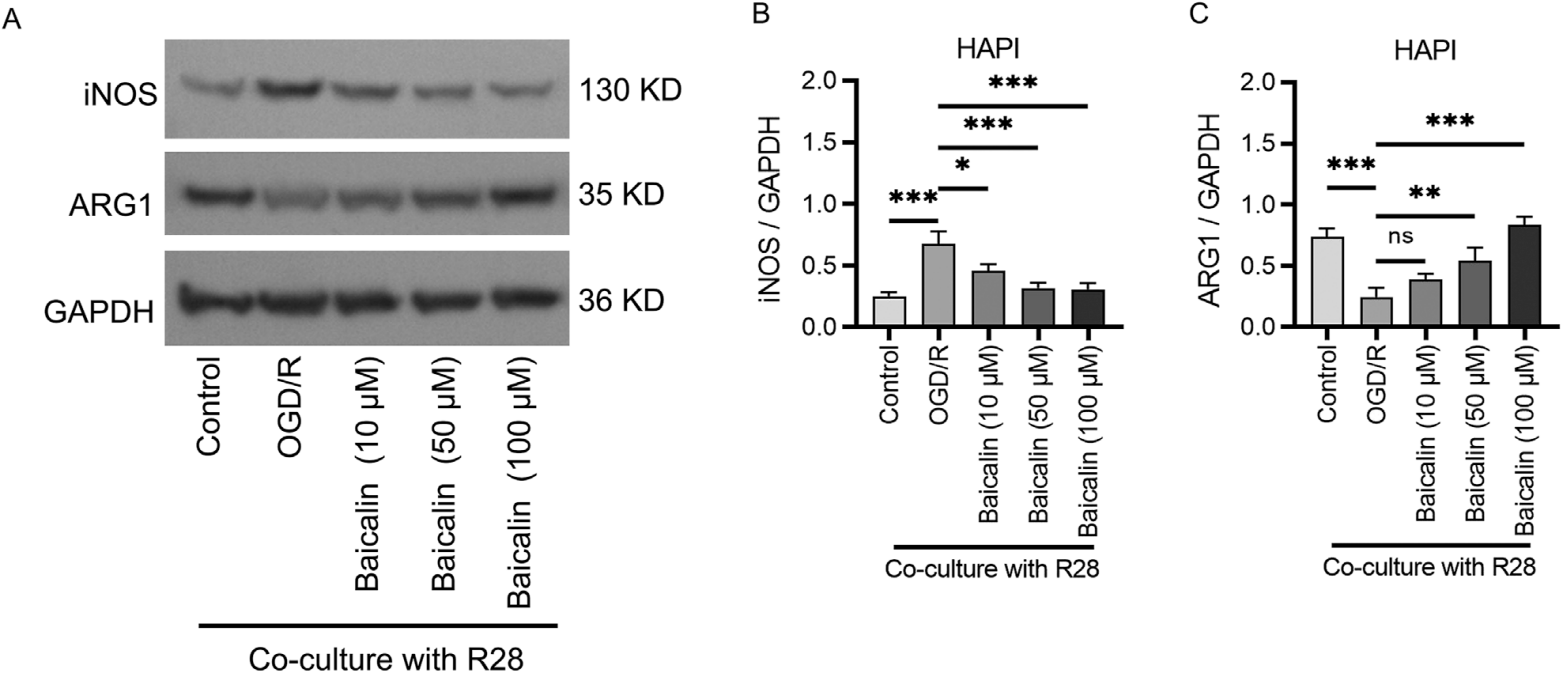
Baicalin modulates microglial polarization. **(A,B)** Expression of the M1 marker iNOS. **(A,C)** Expression of the M2 marker Arg-1. Mean ± SD; *n* = 3/group; *P < 0.05, **P < 0.01, ***P < 0.001. One-way analysis of variance. iNOS, inducible nitric oxide synthase; Arg-1, arginase 1.

To further evaluate the above results, we performed Western blot analysis, which revealed that the expression of the M1 marker iNOS was significantly upregulated (Figures 4A,B), while the expression of the M2 marker Arg-1 was significantly downregulated in the OGD/R group (Figures 4A,C). These results indicated that ischemia-reperfusion activates inflammatory responses in RGCs, and is associated with M1/M2 polarization. In contrast, the baicalin treatment groups showed significantly reduced iNOS expression and significantly increased Arg-1 expression, as compared to the OGD/R group, with the most pronounced changes observed in the 100 μM group. This suggests that baicalin regulates the balance between M1 and M2 polarization of the microglia.

### Baicalin regulates the JAK2/STAT3 pathway

**FIGURE 5:**
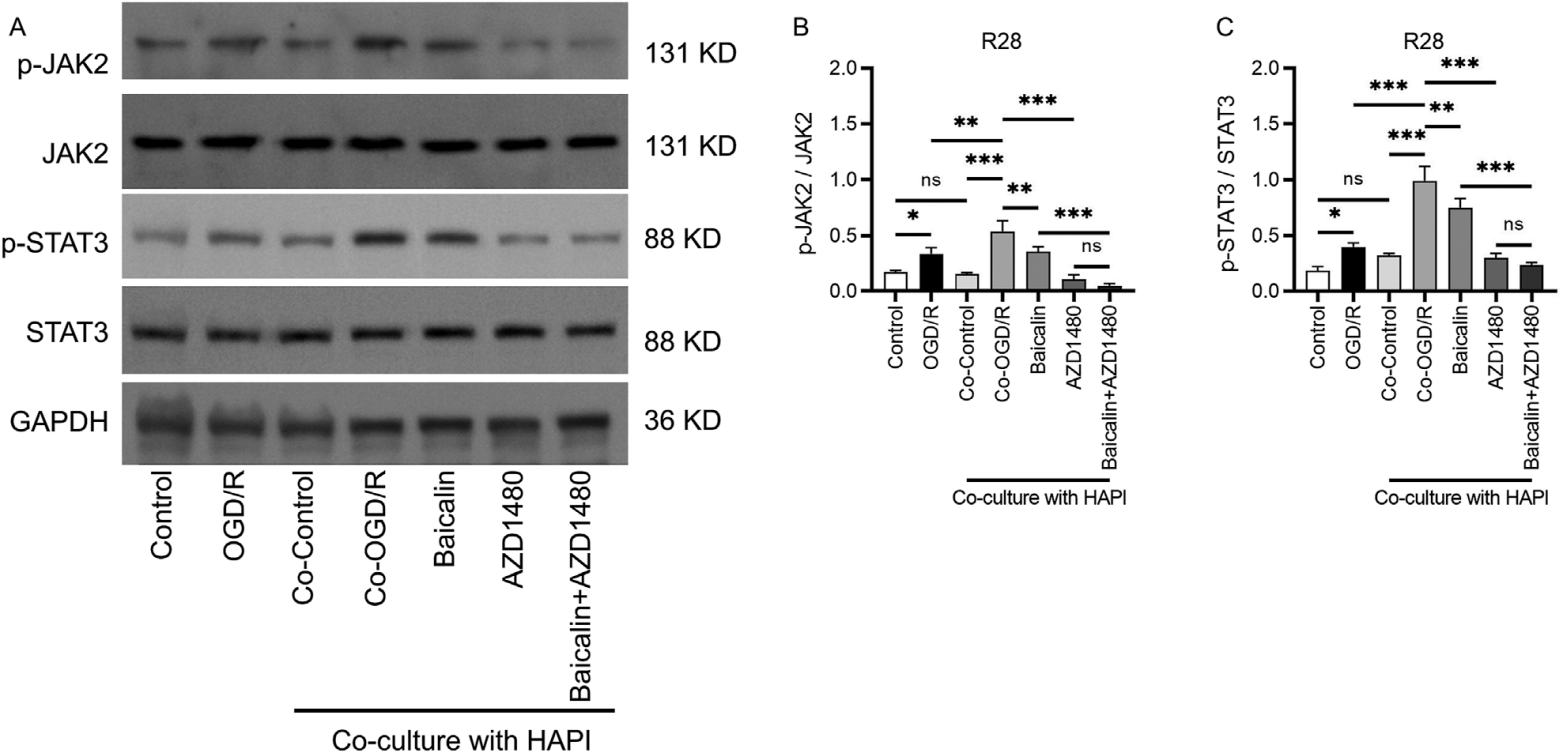
Baicalin regulates the JAK2/STAT3 signaling pathway. **(A–C)** Phosphorylation levels of JAK2/STAT3 in the normal control group, OGD/R group, Co-Control group, Co-OGD/R group, baicalin group, and combined treatment group with baicalin and AZD1480. Mean ± SD; *n* = 3/group; *P < 0.05, **P < 0.01, ***P < 0.001. One-way analysis of variance. OGD/R, oxygen-glucose deprivation/reperfusion.

Numerous studies have demonstrated the critical role of the JAK2/STAT3 pathway in ischemia-reperfusion injury (Yu et al., 2011; Song et al., 2023; Zhang et al., 2020). We investigated the effects of baicalin on the expression levels of proteins related to the JAK2/STAT3 pathway. Western blot analysis showed that the phosphorylation levels of JAK2/STAT3 were elevated in the OGD/R and co-culture groups, as compared to the control group. This indicated activation of the JAK2/STAT3 signaling pathway under ischemia-reperfusion conditions. After baicalin treatment, the phosphorylation levels of JAK2/STAT3 were significantly reduced as compared to the Co-OGD/R group. When baicalin treatment was combined with the JAK2/STAT3 inhibitor AZD1480, the phosphorylation levels of JAK2/STAT3 were further decreased, suggesting that baicalin inhibits JAK2/STAT3 phosphorylation and exhibits synergistic effects with AZD1480 in suppressing protein phosphorylation in the JAK/STAT3 pathway (Figures 5A–C).

### Impact of JAK2/STAT3 inhibitors on the neuroprotective effects of baicalin

**FIGURE 6:**
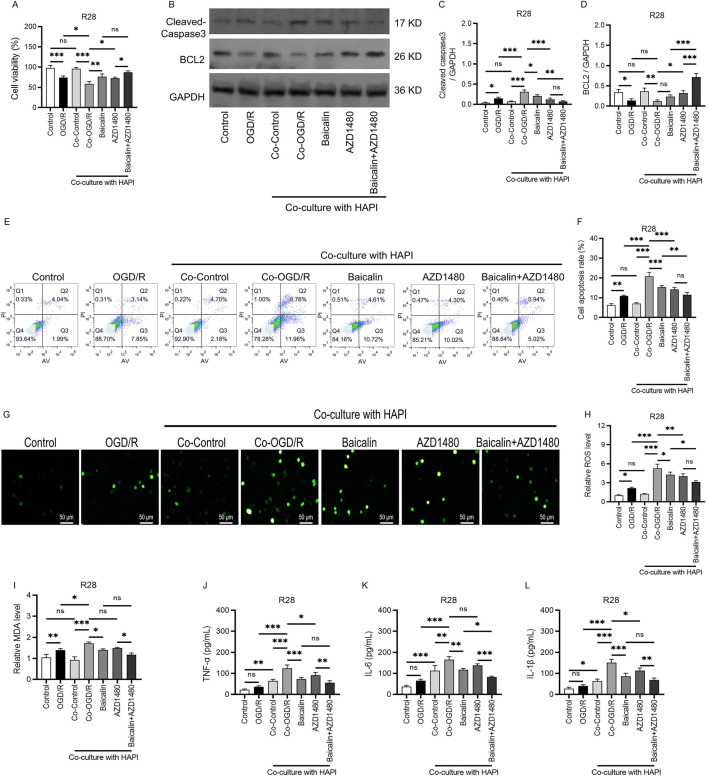
JAK2/STAT3 inhibitor enhances the neuroprotective effects of baicalin. **(A)** Cell viability in different groups. **(B–D)** Expression of the pro-apoptotic protein caspase 3 and anti-apoptotic protein BCL2. **(E,F)** Annexin V/propidium iodide (AV/PI) apoptosis assay results in the normal control group, OGD/R group, Co-Control group, Co-OGD/R group, baicalin group, and combined treatment group with baicalin and AZD1480. **(G,H)** ROS fluorescence intensity in the above groups. Scale bar: 50 μm. **(I)** MDA levels in different groups. **(J–L)** Levels of TNF-α, IL-6, and IL-1β in the seven groups mentioned above. Mean ± SD; *n* = 3/group; *P < 0.05, **P < 0.01, ***P < 0.001. One-way analysis of variance. OGD/R, oxygen-glucose deprivation/reperfusion; ROS, reactive oxygen species; MDA, malondialdehyde; TNF-α, tumor necrosis factor-α; IL, interleukin.

To further validate the role of the JAK2/STAT3 signaling pathway in baicalin-mediated neuroprotection, we used the specific JAK/STAT inhibitor AZD1480. CCK-8 and annexin V/propidium iodide apoptosis assays showed that cell viability was higher in the baicalin + AZD1480 group than in the Co-OGD/R and baicalin groups; cell viability increased by 45% and 14.6%, respectively, indicating that inhibiting the JAK2/STAT3 pathway enhances cell viability. When baicalin was combined with AZD1480, cell viability was also higher than that in the Co-OGD/R and baicalin groups, suggesting that AZD1480 enhances the protective effects of baicalin on cell viability (Figures 6A,E,F). Furthermore, after combined treatment, the caspase 3 activity decreased to 0.072 ± 0.020, which was 65% lower than that in the baicalin group. Additionally, the level of the anti-apoptotic protein BCL2 increased to 0.720 ± 0.083, which was 200% higher than that in the baicalin group (Figures 6B–D). The fluorescence intensity of ROS decreased by 26.5% as compared to the baicalin group (*P* = 0.038), and by 22.3% as compared to the AZD1480 group (*P* = 0.047; Figures 6G,H). The MDA level decreased by 15.9% as compared to the baicalin group (*P* = 0.042), and by 20.9% as compared to the AZD1480 group (*P* = 0.031; Figure 6I), indicating alleviation of oxidative stress damage. The ELISA results showed that compared with the baicalin group, the combined treatment group had significantly reduced expression levels of inflammatory factors (TNF-α, IL-6, and IL-1β), with decreases of 14.3%, 20.9%, and 11.9%, respectively (*P* < 0.001; Figures 6J–L). Collectively, the above results indicate that the JAK2/STAT3 signaling pathway plays a critical role in baicalin-mediated neuroprotection and that the JAK2/STAT3 inhibitor AZD1480 enhances the protective effects of baicalin against the ischemia-reperfusion injury of RGCs by synergistically suppressing JAK2/STAT3 activation.

### Baicalin effectively alleviates ferroptosis

**FIGURE 7:**
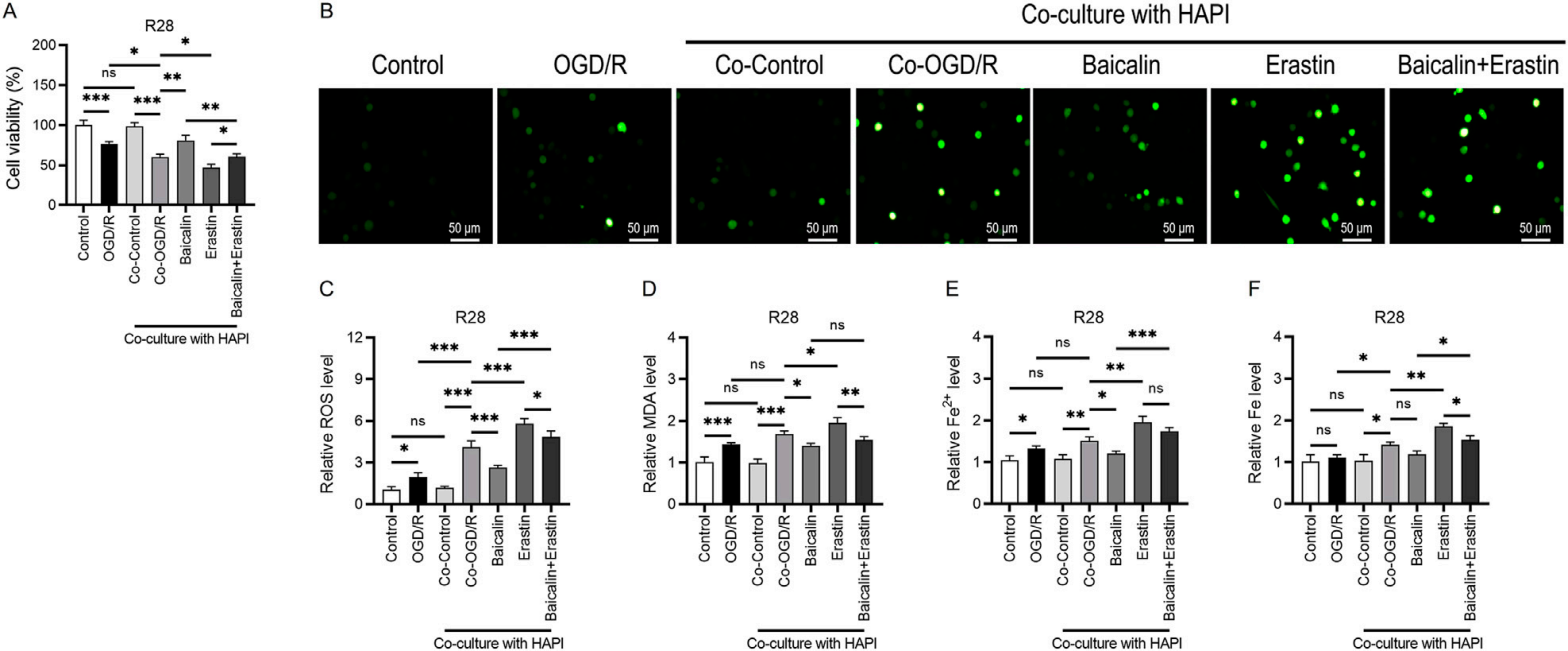
Baicalin effectively attenuates ferroptosis. **(A)** OD value in the Co-OGD/R group, baicalin-alone group, erastin-alone group, and combined treatment group with baicalin and erastin. **(B,C)** ROS fluorescence intensity in the above groups. Scale bar: 50 μm. **(D)** MDA levels in the above groups. **(E,F)** Fe and Fe^2+^ concentrations in the seven groups mentioned above. Mean ± SD; *n* = 3/group; *P < 0.05, **P < 0.01, ***P < 0.001. One-way analysis of variance. ROS, reactive oxygen species; MDA, malondialdehyde.

RGC ferroptosis is a key mechanism of retinal injury and plays a critical role in the pathogenesis of glaucoma (Huang et al., 2023). To further explore the regulatory effects of baicalin on ferroptosis, we used the ferroptosis inducer erastin. CCK-8 assay results showed that the OD value was higher in the baicalin-alone group than in the Co-OGD/R group (Figure 7A). After treatment with erastin, cell viability decreased. When baicalin was combined with erastin, cell viability was higher than in the erastin-alone group, preliminarily indicating that baicalin mitigates erastin-induced cytotoxicity. Additionally, ROS fluorescence probe assays and MDA level measurements showed that ROS and MDA levels were higher in the erastin group than in the Co-OGD/R group, indicating exacerbated oxidative stress damage. When baicalin was combined with erastin, ROS fluorescence intensity and MDA levels were higher than those in the baicalin-alone group but lower than those in the erastin group, suggesting that baicalin inhibits erastin-induced ROS production (Figures 7B–D).

**FIGURE 8:**
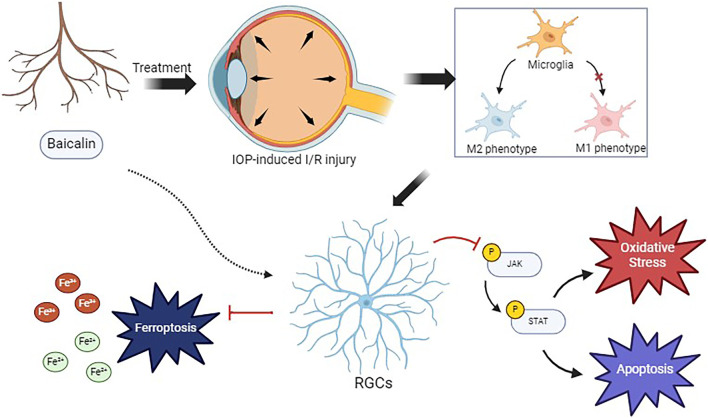
The mechanism underlying the protective effects of baicalin on retinal ganglion cells.

Furthermore, to investigate the regulatory effects of baicalin on ferroptosis, we measured the Fe and Fe^2+^ concentrations. The OGD/R group showed higher Fe and Fe^2+^ concentrations than the control group, indicating that OGD/R treatment increases intracellular Fe and Fe^2+^ levels. The Co-OGD/R group also exhibited higher Fe and Fe^2+^ concentrations than the Co-Control group. In contrast, Fe and Fe^2+^ concentrations were significantly lower in the baicalin group than in the Co-OGD/R group, while the erastin group showed markedly elevated Fe and Fe^2+^ levels as compared to the Co-OGD/R group. However, when baicalin was combined with erastin, the Fe and Fe^2+^ concentrations were significantly reduced, as compared to the erastin group (Figures 7E,F), suggesting that baicalin may reverse erastin-induced ferroptosis by regulating intracellular iron ion dynamics. Taken together, our findings reveal that baicalin markedly inhibits the M1 polarization of microglia, facilitates their transition toward M2 polarization, and mitigates oxidative stress and ferroptosis in RGCs by activating the Nrf2 pathway and suppressing the JAK2/STAT3 signaling pathway (Figure 8).

## Discussion

Glaucoma is characterized by progressive damage to the RGCs and vision loss, with ischemia-reperfusion injury of the RGCs caused by high IOP being a significant pathological factor. Microglial activation and polarization also play a key role in RGC damage, driving glaucoma progression (Salk et al., 2024). While activated microglia can clear dead RGCs and release neurotrophic factors for neuroprotection, their chronic activation and M1 polarization lead to the release of inflammatory factors, worsening inflammation and inducing RGC apoptosis (Luo et al., 2023). This study used an OGD/R-treated microglia-RGC co-culture system and found that baicalin reshapes the neuroimmune microenvironment and regulates iron metabolism, blocking JAK2/STAT3-mediated ferroptosis cascades in the microglia-neuron interaction system.

Our data showed that under ischemia-reperfusion conditions, the pro-inflammatory M1 marker iNOS; the inflammatory factors TNF-α, IL-6, and IL-1β; and the oxidative stress indicators ROS and MDA were upregulated. This indicated that OGD/R drove microglia to pro-inflammatory M1 polarization, releasing inflammatory factors and excessive ROS, exacerbating lipid peroxidation, and increasing RGC ferroptosis sensitivity. Baicalin upregulated the anti-inflammatory M2 marker Arg-1, promoting M1-to-M2 polarization of the microglia. ELISA showed decreased TNF-α, IL-6, and IL-1β after baicalin treatment, suggesting an anti-inflammatory microenvironment. This phenotypic shift reduced neuronal ROS and MDA levels, alleviating lipid peroxidation and blocking ferroptosis triggers.

STAT3 activation inhibits acyl Co-A synthetase long-chain fatty acid member 4 (ACSL4), a lipase rich in long-chain polyunsaturated fatty acids essential for ferroptosis (Luo et al., 2022). Furthermore, studies have shown that inhibiting the excessive activation of JAK2/STAT3 is an effective neuroprotective strategy in central nervous system diseases (Chang et al., 2018; Han et al., 2020). In this study, OGD/R injury triggered inflammatory and oxidative stress responses in the co-culture system of microglia and RGCs, activated the microglia and caused them to polarize towards the pro-inflammatory M1 phenotype, and released a large amount of inflammatory factors and ROS, thereby activating the JAK2/STAT3 pathway, increasing the phosphorylation level of JAK2/STAT3, and exacerbating the inflammatory response and cell death. Baicalin significantly suppressed JAK2/STAT3 phosphorylation and promoted the M1-to-M2 polarization of microglia, highlighting the role of the JAK2/STAT3 pathway in the anti-inflammatory effects of baicalin. Excessive STAT3 activation can cause the nuclear translocation of nuclear factor-κB, upregulating M1 markers and pro-inflammatory factors (Bisserier et al., 2020; Shi et al., 2021). Baicalin blocked this cascade by inhibiting JAK2/STAT3 phosphorylation and activating Arg-1 transcription, enhancing anti-inflammatory factor secretion and providing new insights for the regulation of RGC inflammation.

Ferroptosis, a type of regulated cell death linked to Fe^2+^ accumulation, occurs when glutathione is depleted and glutathione peroxidase 4 (GPX4) activity declines (Bersuker et al., 2019; Liang et al., 2023). In this scenario, lipid oxidants cannot be metabolized via the GPX4-catalyzed glutathione reductase reaction, and Fe^2+^-induced lipid oxidation produces ROS, driving ferroptosis (Jiang et al., 2021). Baicalin effectively reduced intracellular free iron, ROS, and MDA levels, indicating that it inhibits ferroptosis by regulating iron metabolism-related genes and proteins, possibly by specifically suppressing the transferrin receptor TfR1 and promoting the iron-storage protein FTH1 (Kang et al., 2024; Zeng et al., 2021). Compared to conventional ferroptosis inhibitors like ferrostatin-1, this upstream iron metabolism-targeting strategy can interrupt ferroptosis earlier. Fan et al. found that baicalin primarily regulates Tfr1 in an ischemia-reperfusion injury model (Fan et al., 2021), suggesting tissue-specific iron metabolism regulation by baicalin and offering new ideas for organ-targeted drug development.

The ferroptosis regulation mediated by baicalin synergizes with its JAK2/STAT3 pathway inhibition. STAT3, which regulates cellular iron uptake and storage proteins, when over-activated, upregulates TfR1, promoting iron uptake and lipid peroxidation (Cheng et al., 2020; Zheng et al., 2022). Baicalin reduces TfR1 expression and upregulates FTH1 by inhibiting STAT3 phosphorylation, lowering intracellular free iron. The present study showed that baicalin suppressed JAK2/STAT3 activation and significantly reduced Fe^2+^ concentrations after OGD/R injury, indicating potentially reduced iron overload risk. This cross-regulation allows baicalin to target inflammation and oxidative stress simultaneously, offering broader neuroprotection than single-pathway inhibitors.

Our preliminary studies (Yu et al., 2024) have demonstrated the protective effects of baicalin on RGCs in animal experiments. This current study reveals a novel mechanism by which baicalin regulates the microglial-ferroptosis interplay through the JAK2/STAT3 pathway in a glaucoma model. However, currently available data on baicalin derive from *in vitro* co-culture models. Future studies should construct high IOP animal models to verify the effects of baicalin on microglial polarization and ferroptosis, and assess the long-term safety and retinal permeability of combined treatments. Additionally, it is crucial to improve our understanding of the individual effects of baicalin on microglia, and further clarify the direct effects of baicalin on microglia as well as its indirect regulatory effects through other cells.

In summary, the protective effects of baicalin against ischemia-reperfusion injury of the RGCs are achieved through multiple pathways and targets, mainly by inhibiting JAK2/STAT3 activation, alleviating inflammation, regulating iron homeostasis, and enhancing antioxidant capacity. In the microglia-neuron interaction environment, baicalin modulates RGC survival and function via the JAK2/STAT3 pathway, reduces indirect inflammatory damage to the RGCs by regulating microglial polarization, and inhibits ferroptosis, thereby providing systematic protection to RGCs. These findings offer new therapeutic insights and targets for RGC-related ischemia-reperfusion injuries. Future research can further explore the molecular mechanisms underlying the effects of baicalin, and optimize its delivery methods and dosing for clinical application, bringing new hope to retinal disease patients.

## Data Availability

The original contributions presented in the study are included in the article/Supplementary Material, further inquiries can be directed to the corresponding authors.
